# Enhancing Pain Relief in Temporomandibular Joint Arthrocentesis: Platelet-Rich Plasma and Hyaluronic Acid Synergy

**DOI:** 10.7759/cureus.45646

**Published:** 2023-09-20

**Authors:** Akshita N Parlawar, Bhushan P Mundada

**Affiliations:** 1 Dentistry, Sharad Pawar Dental College and Hospital, Datta Meghe Institute of Higher Education and Research, Wardha, IND; 2 Oral and Maxillofacial Surgery, Sharad Pawar Dental College and Hospital, Datta Meghe Institute of Higher Education and Research, Wardha, IND

**Keywords:** regenerative therapy, functional improvement, pain management, arthrocentesis, hyaluronic acid, platelet rich plasma, tmj disorders, temporomandibular joint

## Abstract

Temporomandibular joint (TMJ) disorders present complex challenges in pain management and functional restoration. This review delves into the innovative approach of using platelet-rich plasma (PRP) and hyaluronic acid (HA) combination therapy in TMJ arthrocentesis to address these issues. The potential benefits of this approach are highlighted through an exploration of mechanisms, clinical studies, safety considerations, and future directions. PRP's regenerative properties and HA's lubrication and anti-inflammatory effects offer a comprehensive solution to multifactorial TMJ pain and dysfunction. Clinical studies reveal significant pain reduction, improved mobility, and enhanced satisfaction in patients treated with PRP and HA. Although mild and transient adverse effects have been reported, the safety profile remains favorable. While the evidence is promising, more extensive randomized controlled trials are needed to establish sustained efficacy and safety. As research evolves, collaborative efforts among clinicians and researchers are crucial in realizing the potential of PRP and HA combination therapy, ultimately providing a novel pathway to alleviate TMJ-related pain and enhance patient well-being.

## Introduction and background

Temporomandibular joint (TMJ) disorders encompass a range of conditions affecting the temporomandibular joint, which connects the jawbone to the skull. These disorders can lead to debilitating symptoms such as pain, restricted jaw movement, and discomfort while chewing or speaking. TMJ disorders significantly impact affected individuals' quality of life, contributing to physical and psychological distress [1,2].

Arthrocentesis, a minimally invasive procedure involving needle insertion into the TMJ, has emerged as a valuable tool in managing TMJ disorders. This procedure aims to alleviate pain, improve joint mobility, and reduce inflammation by flushing debris and inflammatory byproducts from the joint space. As a minimally invasive technique, arthrocentesis offers advantages such as rapid recovery and lower complication rates than more invasive surgical interventions [3,4].

Recent developments in regenerative medicine have led to exploring platelet-rich plasma (PRP) and hyaluronic acid (HA) as potential adjunctive therapies in TMJ arthrocentesis. PRP, a concentrated solution of platelets derived from the patient's blood, is rich in growth factors and cytokines that play a crucial role in tissue healing and regeneration. Hyaluronic acid, a natural component of synovial fluid, has lubricating properties and anti-inflammatory effects, making it a promising candidate for alleviating pain and promoting joint health [5,6].

This review aims to consolidate the evidence regarding using PRP and HA in combination for pain reduction in TMJ arthrocentesis. By examining the outcomes of relevant clinical studies, mechanistic insights, and potential synergistic effects, this paper seeks to provide a comprehensive assessment of the therapeutic potential of PRP and HA in improving the outcomes of TMJ arthrocentesis procedures. This review aims to shed light on this combined approach's feasibility, safety, and efficacy, ultimately contributing to optimizing pain management strategies for individuals suffering from TMJ disorders.

## Review

Platelet-rich plasma (PRP) and therapeutic applications

Platelet-rich plasma (PRP) is a biological product derived from the patient's blood, enriched with a higher concentration of platelets than typically found in whole blood. Platelets are crucial blood components responsible for wound healing and tissue repair. PRP contains a range of growth factors, cytokines, and bioactive molecules that are pivotal in the body's natural healing processes [7].

PRP has garnered considerable attention across diverse medical disciplines, encompassing orthopedics, dermatology, and dentistry, primarily due to its remarkable capacity for augmenting tissue regeneration and fostering healing. In orthopedics, PRP injections have emerged as an invaluable approach for addressing various conditions, including tendon injuries, osteoarthritis, and muscle strains. Researchers have demonstrated that the rich growth factors within PRP formulations play a pivotal role in stimulating cell proliferation, facilitating collagen synthesis, and promoting angiogenesis. These findings collectively underscore PRP's pivotal role in driving tissue repair and regeneration, substantiating its effectiveness in clinical practice [8].

Role of hyaluronic acid (HA) in joint health and medical treatments

Hyaluronic acid (HA) is a glycosaminoglycan that naturally occurs in synovial fluid, acting as a lubricant and cushion for joints. This vital component of connective tissues plays a pivotal role in preserving joint health and mobility. Research findings have demonstrated the efficacy of HA in various patient groups, providing insights into its age-specific benefits and frequency of administration. HA has gained widespread recognition in clinical practice for its effectiveness in managing osteoarthritis, particularly in weight-bearing joints like the knee. Studies involving patients across different age groups have consistently shown positive outcomes. For instance, a study conducted on individuals aged 45 to 65 reported a significant reduction in joint pain and improved mobility following the administration of HA [9]. Moreover, research has revealed that the frequency of HA injections can vary based on individual needs, with some patients benefiting from monthly treatments. In contrast, others find relief with less frequent administration [10]. These findings underscore the versatility of HA as an injectable therapy for osteoarthritis, highlighting its ability to enhance joint lubrication, reduce inflammation, and alleviate pain in patients of varying age groups.

Challenges in pain management for TMJ disorders

Temporomandibular joint (TMJ) disorders pose unique challenges for pain management. The complex anatomy of the TMJ, coupled with the multifactorial nature of TMJ disorders, often results in varying degrees of pain and dysfunction. Conventional pain management approaches include nonsteroidal anti-inflammatory drugs (NSAIDs), physical therapy, and occlusal splints. However, these methods may not always provide satisfactory relief, and more invasive interventions like surgery are considered only when conservative options fail [11].

The temporomandibular joint's frequent movement and exposure to mechanical stress add to the difficulty in achieving effective and sustained pain relief. Additionally, individual responses to pain medications and treatments vary, making establishing a standardized approach to pain management in TMJ disorders challenging. As a result, there is a need for innovative and tailored interventions that address the underlying mechanisms of pain and inflammation in the TMJ, making the exploration of therapies like PRP and HA combination particularly relevant [12].

Mechanisms of action

PRP's Role in Tissue Regeneration and Pain Reduction

Platelet-rich plasma (PRP) exerts its therapeutic effects through its rich content of growth factors, cytokines, and bioactive molecules. When PRP is introduced into damaged or inflamed tissues, these factors stimulate cell migration, proliferation, and differentiation. Growth factors like platelet-derived growth factor (PDGF), transforming growth factor-beta (TGF-β), and vascular endothelial growth factor (VEGF) promote angiogenesis, collagen synthesis, and tissue remodeling [8].

In tissue regeneration, PRP promotes the recruitment of stem and progenitor cells to the injury site, facilitating tissue repair and regeneration. PRP’s anti-inflammatory properties also help modulate the immune response, reducing inflammation and pain. The overall effect is a more efficient healing process, potentially reducing pain perception [13].

Hyaluronic Acid's Contributions to Joint Health and Pain Relief

Hyaluronic acid (HA) is critical in joint health due to its unique viscoelastic properties. HA acts as a lubricant in the synovial fluid of joints, reducing friction between articular surfaces during movement. This lubrication enhances joint mobility and reduces wear and tear on the joint components [14]. Moreover, HA exhibits anti-inflammatory effects by interacting with cell surface receptors and modulating immune responses. This anti-inflammatory action helps mitigate the local inflammatory processes contributing to joint disorders' pain and discomfort. HA also provides a cushioning effect, absorbing shock and distributing mechanical loads more evenly across the joint, reducing pain [15].

Synergistic Effects of PRP and HA Combination

The combination of platelet-rich plasma (PRP) and hyaluronic acid (HA) holds promise due to their complementary mechanisms of action. PRP's regenerative properties can enhance tissue healing and repair, while HA's lubrication and anti-inflammatory effects contribute to joint comfort and pain reduction. Using PRP and HA together may result in synergistic effects that optimize pain relief and functional improvement in TMJ disorders [16].

PRP's growth factors and cytokines could amplify the anti-inflammatory effects of HA, promoting a more balanced and controlled immune response within the joint. Simultaneously, HA's lubricating properties could enhance the distribution of PRP's healing factors within the joint space, potentially prolonging their beneficial effects [17].

Furthermore, it is crucial to elucidate the timing and dosage considerations for the combined treatment's efficacy. We can better understand the cumulative impact on pain perception by considering the optimal administration schedule and dosage levels for both PRP and HA. This combination therapy addresses various aspects of pain generation, including inflammation, tissue damage, and impaired lubrication. Consequently, it may provide a more comprehensive approach to pain reduction than individual treatments alone [18].

Combination drug therapies, such as the fusion of PRP and HA, are employed in regenerative medicine and joint pain management, catering to diverse medical conditions. The precise dosage, treatment duration, and administration procedures are contingent on factors such as the patient's condition and the physician's advice. PRP dosages are calibrated relative to the volume of blood extracted and the desired platelet concentration, typically aiming for a five- to 10-fold increase over normal levels. Typically, PRP is administered at volumes ranging from 3 to 5 milliliters per targeted site. In the case of HA, the dose hinges on the specific product and treatment site, with usual injections spanning 1 to 3 milliliters per joint or focal point. The treatment regimen typically consists of a series of injections, commonly totaling three to five sessions distributed over one to two-week intervals. Subsequent booster injections may be necessary for sustained pain relief and joint function, the timing of which can range from several months to a year or more. These injections are typically administered directly into the affected area under the guidance of a qualified healthcare provider, frequently employing ultrasound or fluoroscopy for precision. The potential synergy between PRP and HA warrants further investigation through clinical studies that evaluate their combined effects on pain relief, functional outcomes, and patient satisfaction. Such research could provide valuable insights into the optimal dosing, timing, and application techniques to maximize the benefits of this combined approach in TMJ arthrocentesis procedures [19].

Safety and adverse effects

Safety Profile of PRP and HA Combination for TMJ Arthrocentesis

Based on the available literature, the safety profile of using platelet-rich plasma (PRP) and hyaluronic acid (HA) in combination for TMJ arthrocentesis appears favorable. PRP and HA are derived from the patient's body, so the risk of allergic reactions or immune responses is minimal. Moreover, the minimally invasive nature of arthrocentesis contributes to reduced risks compared to more invasive procedures [20].

Studies have reported no serious adverse events directly attributed to the PRP and HA combination. The autologous nature of these treatments adds a layer of safety, as the risk of disease transmission or rejection is virtually eliminated. However, healthcare practitioners must adhere to proper aseptic techniques during preparation and administration to minimize infection risk [21-25].

Potential Adverse Effects

While the utilization of platelet-rich plasma (PRP) and hyaluronic acid (HA) combination therapy for temporomandibular joint (TMJ) disorders is generally considered safe, it's essential to acknowledge that, like any medical intervention, there are potential adverse effects. However, they are usually mild and transient. Serious adverse effects are rare and have not been widely reported [26].

Mild discomfort and swelling at the injection site: In a minority of cases, patients might experience mild discomfort or swelling at the injection site. This is common with various injectable therapies and is often attributed to the physical presence of the injected substances within the joint space. However, these reactions are typically self-limiting and tend to resolve within a short period of time without requiring specific medical intervention [27].

Inflammatory reactions: Occasionally, patients may exhibit mild inflammatory reactions following the introduction of PRP and HA into the TMJ. These reactions might manifest as localized redness or warmth around the injection site. While our review did not uncover specific reports detailing which drug led to a higher incidence of adverse effects, whether there were significant differences in adverse effects after the administration of both drugs or if changing the schedule of administration influenced these reactions, it is an important aspect to consider for future research. It's crucial to note that these reactions often indicate the body's natural response to introducing foreign substances and are part of the healing process. Such reactions are generally short-lived and well-tolerated by patients [28].

It's crucial to emphasize that adverse effects can vary from individual to individual, and some patients may not experience any adverse effects at all. Healthcare practitioners must exercise caution and adhere to proper administration techniques to minimize the risk of adverse reactions. Moreover, patients should be informed about the potential for these mild and transient adverse effects as part of the informed consent process [29].

Importance of Patient Selection and Administration Techniques

Proper patient selection is paramount to ensuring the safety and effectiveness of PRP and HA combination therapy for TMJ arthrocentesis. This entails a meticulous assessment of patient eligibility, including age-related factors. Patients should be thoroughly evaluated to identify contraindications, such as bleeding disorders, active infections, or allergies. Furthermore, a comprehensive review of the patient's medical history, current medical conditions, and medications should be conducted to minimize potential risks [30].

Additionally, the administration technique plays a crucial role in minimizing risks. Proper injection techniques, sterile equipment, and adherence to aseptic protocols are essential to prevent infection and other complications. Precise placement of the injections within the TMJ space is critical to ensure optimal distribution of PRP and HA and to avoid injury to surrounding structures [31].

Healthcare practitioners should remain vigilant in monitoring patients who have undergone PRP and HA administration during TMJ arthrocentesis. It is important to consider the duration of this monitoring, as it allows for the early identification and management of potential adverse effects. Like other medical procedures, ensuring comprehensive patient education and obtaining informed consent is crucial to enable patients to fully comprehend the potential benefits and risks associated with the treatment [32].

Mechanistic insights

Cellular and Molecular Pathways of PRP and HA Combination

The pain-reducing effects of combining platelet-rich plasma (PRP) and hyaluronic acid (HA) within TMJ arthrocentesis involve intricate and interconnected cellular and molecular pathways. This innovative therapeutic approach capitalizes on the unique properties of PRP and HA to address multiple aspects of TMJ disorders, ultimately leading to pain relief and functional improvement [5].

Inflammation modulation: PRP's composition, rich in growth factors, offers the potential to regulate the inflammatory response within the TMJ. The presence of anti-inflammatory cytokines within PRP may counteract pro-inflammatory signals, contributing to the reduction of local inflammation. By shifting the balance towards an anti-inflammatory environment, PRP can alleviate pain associated with inflammation within the joint [33].

Tissue regeneration: PRP's growth factors are pivotal in stimulating cellular activities such as migration and proliferation. These processes are essential for tissue repair and regeneration. When introduced into the TMJ, PRP may facilitate the recruitment of reparative cells, which can aid in healing damaged tissues when combined with HA's lubricating effects. This dual action may decrease pain by addressing the underlying tissue damage contributing to discomfort [34].

Neurological impact: PRP's growth factors also potentially influence pain perception through neuroregulation. By interacting with pain signaling pathways and nerve tissues, PRP could modulate the transmission of pain signals. Concurrently, HA's anti-inflammatory effects could further reduce nerve sensitization, potentially leading to a dampened pain response. This combined influence on pain perception may contribute to overall pain reduction in individuals with TMJ disorders [35].

Lubrication enhancement: HA's inherent properties as a lubricant are particularly relevant to the joint's mechanical function. By enhancing joint lubrication and reducing friction between surfaces, HA promotes smoother movement within the TMJ. This, in turn, eases mechanical stress and minimizes the generation of pain during jaw movement. Improved lubrication also enhances joint mobility, which is essential for overall functional improvement [36].

Synergistic Mechanism of Action

While limited histological and biochemical evidence directly supporting the synergistic mechanism of PRP and HA in TMJ arthrocentesis exists, the available data from related fields provide insights. HA has been shown to support cartilage integrity and protect against degradation in joint conditions like osteoarthritis. PRP, on the other hand, accelerates tissue regeneration and has anti-inflammatory effects [37].

In combination, PRP could enhance HA's protective effects on cartilage by promoting chondrocyte viability and collagen synthesis. HA's lubrication and anti-inflammatory actions might facilitate the distribution and persistence of PRP's healing factors within the joint space. This interaction could lead to a more comprehensive attenuation of pain and improvement in joint function than either treatment alone [38]. Further studies are needed to provide direct histological and biochemical evidence of this synergistic interaction in the context of TMJ disorders. Exploring gene expression patterns, cytokine profiles, and cellular responses within the TMJ following PRP and HA treatment could illuminate the combined effects and potential underlying mechanisms.

Clinical implications

Potential Clinical Benefits of PRP and HA in TMJ Arthrocentesis

The utilization of platelet-rich plasma (PRP) and hyaluronic acid (HA) in TMJ arthrocentesis procedures represents a promising advancement in the field of temporomandibular joint (TMJ) disorder management. This innovative combination therapy capitalizes on PRP and HA's distinct yet complementary properties, leading to a comprehensive approach with significant clinical promise for individuals struggling with TMJ-related pain and dysfunction [6].

Enhanced pain relief and duration: One of the notable strengths of PRP and HA combination therapy is its potential to provide enhanced pain relief compared to conventional treatments, with research findings indicating its effectiveness in addressing pain duration and anesthetic effects. This synergistic interplay between PRP's regenerative and anti-inflammatory properties and HA's lubricating and pain-relieving effects allows the treatment to address pain from multiple angles. Studies have demonstrated that this combination therapy alleviates pain and provides insights into how long it takes to relieve pain. Research has shown that PRP promotes tissue healing and reduces inflammation, leading to rapid pain relief within a relatively short timeframe. Additionally, HA's capacity to alleviate mechanical stress through lubrication complements the pain relief effects of PRP. Studies have reported that the combined therapy's multi-faceted approach to pain management targets various pain pathways, including inflammation, tissue damage, and mechanical stress, thereby offering the potential for superior and sustained pain reduction. Furthermore, research has investigated the potential anesthetic effects of these drugs, shedding light on their impact on pain perception and sensory nerve responses in the context of TMJ disorder patients [39]. This holistic approach not only improves pain relief but also enhances the overall quality of life for individuals suffering from TMJ disorders.

Functional improvement: Another compelling aspect of PRP and HA combination therapy is its potential to facilitate functional improvement in individuals with TMJ disorders. PRP's regenerative effects come into play here, as it may stimulate tissue repair and regeneration within the TMJ. This could restore joint function and address restricted jaw movement and discomfort during daily activities such as eating and speaking. HA’s lubricating properties also play a crucial role in enhancing joint mobility and reducing friction during movement. This synergy between PRP and HA promises to improve overall joint function and enhance the quality of life for individuals with TMJ disorders [6].

Reduced need for medication: The successful implementation of PRP and HA combination therapy offers effective pain relief for TMJ disorder patients and presents a compelling alternative to traditional pain medications such as NSAIDs and steroids. This combination therapy holds the potential to significantly reduce the reliance on analgesic drugs, which is of paramount importance given the potential side effects associated with prolonged use of these medications. By addressing the underlying factors contributing to pain, PRP and HA therapy improve patient comfort and mitigate the potential risks and side effects linked to extended drug use. This approach aligns with a holistic and patient-centered approach to TMJ disorder management [6].

Relevance of the Approach in Improving Patient Outcomes

Minimally invasive procedure: TMJ arthrocentesis is inherently minimally invasive, aligning seamlessly with contemporary patient preferences for less-invasive treatments involving shorter recovery times. PRP and HA in this context further enhance this advantage, as their application via injections avoids the need for extensive surgical incisions or invasive interventions [33].

Customized treatment approach: One of the remarkable attributes of PRP and HA combination therapy is its adaptability to cater to each patient's needs. This personalized treatment approach considers factors such as the severity of the condition, the patient's medical history, and specific pain patterns. This tailoring ensures the therapy is optimized for each patient, potentially yielding more effective pain relief and functional improvement [40].

Holistic treatment strategy: The multi-faceted nature of TMJ disorders necessitates a comprehensive approach that addresses various contributing factors simultaneously. The synergistic interaction between PRP's regenerative properties and HA's lubrication and anti-inflammatory effects exemplifies this holistic strategy. By targeting not only pain but also inflammation, tissue damage, and joint function, this approach has the potential to lead to more thorough and lasting outcomes [41].

Potential for long-term benefits: The regenerative effects associated with PRP introduce the possibility of long-lasting improvements in joint health and function. By promoting tissue repair and regeneration, PRP could result in enduring benefits, reducing the likelihood of recurrent symptoms and diminishing the need for frequent interventions. This aspect significantly impacts the overall quality of life of individuals with chronic TMJ disorders [42].

Future directions

Areas for Further Research and Investigation

Long-term efficacy: The durability of the benefits provided by platelet-rich plasma (PRP) and hyaluronic acid (HA) combination therapy is a critical aspect that requires further investigation. Longitudinal studies with extended follow-up periods are essential to monitor the sustained outcomes of this treatment approach. Understanding how long the pain relief and functional improvements persist after the initial intervention is crucial for determining the necessity and timing of potential repeat treatments. These studies will shed light on whether the benefits of the PRP and HA combination are sustained over time or if there is a need for periodic re-administration. By assessing the long-term efficacy, researchers and clinicians can offer patients more accurate expectations regarding the treatment's lasting impact on their quality of life.

Comparison studies: Comparative studies are imperative to establish the position of PRP and HA combination therapy within the spectrum of TMJ disorder treatments. Conducting head-to-head comparison studies that evaluate the efficacy of this approach against other established treatments, such as corticosteroid injections or traditional arthrocentesis, can provide valuable insights. Such studies allow for a direct comparison of outcomes, including pain reduction, functional improvement, and patient satisfaction. Comparative analyses offer a nuanced understanding of the strengths and limitations of PRP and HA combination therapy and existing options. This information is crucial for clinicians and patients to decide on the most suitable treatment approach based on individual needs and treatment goals.

Mechanistic studies: A deeper understanding of the molecular and cellular mechanisms underpinning the synergistic effects of PRP and HA is essential for advancing our knowledge of this therapeutic approach. In-depth mechanistic studies can elucidate how PRP's regenerative properties interact with HA's lubrication and anti-inflammatory effects at a cellular level. Researchers can uncover the precise ways these therapies achieve pain reduction and functional improvement by investigating gene expression patterns, cytokine profiles, and cellular responses within the TMJ after PRP and HA treatment. Such mechanistic insights can pave the way for developing more targeted and optimized treatment protocols. They can also inform the design of future interventions that leverage the synergistic interactions of these therapies to achieve even more robust and lasting outcomes for patients with TMJ disorders.

Refinements in PRP and HA Combination Techniques

Optimal formulation: Determining the ideal formulation parameters requires consistent and reliable outcomes with platelet-rich plasma (PRP) and hyaluronic acid (HA) combination therapy. This involves identifying the most effective PRP concentration and HA dosage that provide the desired therapeutic effects while minimizing potential adverse reactions. PRP concentration and HA dosage variations might influence their interactions within the joint space, affecting pain reduction, tissue regeneration, and lubrication. Rigorous experimentation and well-designed clinical trials are necessary to establish the optimal formulation that yields the best balance between these factors, ensuring that patients receive the maximum benefits without undue risk.

Injection timing: Investigating the optimal timing for administering PRP and HA following TMJ arthrocentesis is critical to treatment refinement. Understanding the body's natural healing processes and inflammatory responses can guide the timing of injections to maximize therapeutic effects. Administering PRP and HA at a specific post-arthrocentesis time point might enhance their interaction with healing tissues and mitigate inflammation. Aligning the injections with the appropriate healing phases could improve tissue regeneration, reduce pain, and enhance functional outcomes. Robust clinical studies involving different injection timing strategies and longitudinal assessments are necessary to ascertain the ideal temporal sequence for achieving the best results.

Combination protocols: Developing standardized protocols for administering PRP and HA in TMJ arthrocentesis procedures is pivotal for ensuring treatment consistency and reproducibility across different clinical settings. These protocols encompass various aspects, including injection techniques, depth, and TMJ space localization. Standardization helps minimize variability in treatment delivery, ensuring that patients receive uniform therapeutic interventions. Establishing clear guidelines for injection techniques can prevent inadvertent complications, while determining the optimal depth and localization can maximize the distribution and effectiveness of PRP and HA within the joint. Collaboration among clinicians, researchers, and experts in the field is essential to formulate comprehensive and detailed combination protocols that contribute to safer and more effective treatments.

Importance of Larger Randomized Controlled Trials

Statistical power: In the context of evaluating platelet-rich plasma (PRP) and hyaluronic acid (HA) combination therapy, more extensive randomized controlled trials (RCTs) play a crucial role in enhancing the statistical power of the study. Statistical power refers to the ability of a study to detect significant differences or effects if they truly exist. By involving a more significant number of participants, RCTs can provide more robust evidence regarding the efficacy and safety of the treatment. This is particularly important when assessing subtle differences in outcomes, such as variations in pain reduction or functional improvement. The larger the sample size, the more sensitive the study becomes to detecting meaningful changes, thus increasing the reliability of the findings. Therefore, larger RCTs are essential in establishing a solid foundation of evidence for the effectiveness of PRP and HA combination therapy in TMJ disorders.

Diverse patient populations: Conducting RCTs involving diverse patient populations is essential for comprehensively understanding the treatment's applicability and outcomes. TMJ disorders can manifest with varying severity and across different age groups. By including participants with different levels of severity and from different demographic backgrounds, RCTs can provide insights into how PRP and HA combination therapy performs across a range of clinical scenarios. This enhances the generalizability of the findings and allows healthcare practitioners to tailor the treatment approach based on individual patient characteristics. Diverse RCT populations mirror the real-world diversity of patients seeking treatment for TMJ disorders and ensure that the therapy's benefits are evaluated under various circumstances.

Long-term effects: While short-term outcomes are essential indicators of immediate treatment efficacy, the long-term effects of PRP and HA combination therapy are equally significant for determining its lasting benefits. Larger RCTs with extended follow-up periods are essential for assessing the durability of treatment effects over time. Pain reduction, functional improvement, and patient satisfaction should be evaluated immediately post-treatment and months or even years later. This comprehensive evaluation can provide a more accurate understanding of the treatment's sustained impact and show whether the benefits endure over the long term. Additionally, longer follow-ups can help identify potential late-onset adverse effects or complications that may arise after a certain period.

## Conclusions

In conclusion, our comprehensive exploration of platelet-rich plasma (PRP) and hyaluronic acid (HA) combination therapy in the context of temporomandibular joint (TMJ) disorders and arthrocentesis has unveiled a promising avenue for addressing the intricate challenges associated with TMJ-related pain and dysfunction. Throughout this review, we have delved into the mechanisms, clinical studies, safety considerations, and future directions, illuminating the potential of this approach to revolutionize pain management strategies and enhance the quality of life for TMJ disorder patients. When examining the use of PRP, HA, or either of these two drugs individually, it is crucial to consider the specific outcomes achieved. In some cases, using a single drug may have resulted in less favorable outcomes in pain relief and improved TMJ function. The duration and doses of these drugs also play a significant role in determining their effectiveness. Further, understanding the nuanced differences in outcomes associated with single-drug therapy versus the combination of PRP and HA can provide valuable insights for clinicians and researchers alike. The synergistic interplay between PRP's regenerative properties and HA's lubrication and anti-inflammatory effects presents a comprehensive solution to the multi-faceted nature of TMJ disorders. While our analysis of existing evidence is encouraging, it is imperative to acknowledge that more extensive randomized controlled trials and mechanistic studies are essential to validate the long-term efficacy and safety of this therapeutic option. Moreover, providing detailed information about the specific doses and treatment durations employed in clinical studies can aid in understanding the optimal administration of PRP and HA combination therapy. This information is vital for practitioners seeking to implement this approach in their clinical practice and for researchers designing future investigations. As we advance in our pursuit of effective TMJ disorder management, it becomes evident that a collaborative effort between clinicians, researchers, and scientists is paramount. This collective approach will be instrumental in harnessing the full potential of PRP and HA combination therapy, ultimately offering a promising pathway to alleviate the burdens of TMJ-related pain and improve the overall well-being of TMJ disorder patients.
